# Genome Annotation of *Burkholderia* sp. SJ98 with Special Focus on Chemotaxis Genes

**DOI:** 10.1371/journal.pone.0070624

**Published:** 2013-08-05

**Authors:** Shailesh Kumar, Surendra Vikram, Gajendra Pal Singh Raghava

**Affiliations:** Bioinformatics Centre, Council of Scientific and Industrial Research - Institute of Microbial Technology, Sector 39-A, Chandigarh, India; Louisiana State University and A & M College, United States of America

## Abstract

*Burkholderia* sp. strain SJ98 has the chemotactic activity towards nitroaromatic and chloronitroaromatic compounds. Recently our group published draft genome of strain SJ98. In this study, we further sequence and annotate the genome of stain SJ98 to exploit the potential of this bacterium. We specifically annotate its chemotaxis genes and methyl accepting chemotaxis proteins. Genome of *Burkholderia* sp. SJ98 was annotated using PGAAP pipeline that predicts 7,268 CDSs, 52 tRNAs and 3 rRNAs. Our analysis based on phylogenetic and comparative genomics suggest that *Burkholderia* sp. YI23 is closest neighbor of the strain SJ98. The genes involved in the chemotaxis of strain SJ98 were compared with genes of closely related *Burkholderia* strains (i.e. YI23, CCGE 1001, CCGE 1002, CCGE 1003) and with well characterized bacterium *E. coli* K12. It was found that strain SJ98 has 37 *che* genes including 19 methyl accepting chemotaxis proteins that involved in sensing of different attractants. Chemotaxis genes have been found in a cluster along with the flagellar motor proteins. We also developed a web resource that provides comprehensive information on strain SJ98 that includes all analysis data (http://crdd.osdd.net/raghava/genomesrs/burkholderia/).

## Introduction

The genus *Burkholderia* was created in 1992 and presently contains nearly 72 well-characterized species isolated from a wide range of ecological niches including soil, water, human, plant and clinical samples [1], [2], [3]. The ecological versatility of the genus *Burkholderia* has been attributed to two main factors: i) contain an array of insertion sequences and ii) it is metabolically robust due to its large coding capacity [4]. They evolve by increasing their genome size and changing the gene order in the genome [5]. *Burkholderia* degrade many xenobiotic compounds including polycyclic aromatic hydrocarbons, halogenated hydrocarbons (e.g. trichloroethylene, polychlorinated biphenyl compounds) and pesticides [6]. The wide substrate diversity of these bacteria makes them attractive bioremediation agents. *Burkholderia* sp. strain SJ98 (formerly known as *Ralstonia* sp. SJ98 and further characterized as *Burkholderia* sp. strain SJ98) was isolated from a pesticide contaminated soil sample from Assam agricultural fields, India by using an enrichment technique developed by Samanta *et al*. (2000) ‘chemotactic enrichment technique’ [7]. Various *Burkholderia* spp. have been isolated from soil samples for their property to degrade organophosphate pesticides and aromatic compounds. Bacterial chemotaxis, movement under influence of a chemical gradient, is reasonably argued to enhance biodegradation as it increases bioavailability of a pollutant to the bacteria. Strain SJ98 could completely mineralize or co-metabolized the various nitroaromatic compounds (NACs) and chloronitroaromatic compounds (CNACs) and also shows chemotaxis activity towards these compounds [7], [8], [9], [10], [11]. Strain SJ98 shows chemotaxis activity towards only the compounds it degrades or co-metabolically transforms, but it is not chemotactic towards compounds, which it could not degrade or transforms [8]. In past, Parkinson et. al. 2005 and Falke & Hazelbauer 2001 have reported the chemotaxis system of *E. coli* by flagellar movement [12], [13]. Chemotaxis pathway of *E. coli* have 10 genes including 4 methyl accepting chemotaxis proteins (MCPs) and 6 Che proteins, most of which are organized in a cluster near the flagellar genes [14]. Tran *et al*., (2008) has reported that chemotaxis of *Geobacter* spp. involves numerous chemoreceptors and chemotaxis like gene clusters involved in diverse set of signaling function as well as in chemotaxis [15].

Earlier the genome of *Burkholderia* sp. SJ98 was sequenced by Roche’s 454 and the draft genome sequence is available at our web portal [16]. In this study, strain SJ98 genome has been again sequenced to improve the quality of previously assembled genome. Further, annotations have been performed to explore the bioremediation potential of this microbe. We also determined phylogenetic relationship of this microbe with other closely related *Burkholderia* strains. Genes involved in the chemotaxis of strain SJ98 were annotated and compared with the closest neighbor *Burkholderia* strains YI23, CCGE 1001, CCGE 1002 and CCGE 1003.

## Materials and Methods

### DNA Isolation, Genome Sequencing and Assembly

The genomic DNA was isolated from the *Burkholderia* sp. strain SJ98 using Murmur’s DNA isolation technique [17] and was analyzed by agarose gel electrophoresis. Genome of *Burkholderia* sp. SJ98 was sequenced by Illumina GA IIX sequencing platform at Genotypic Pvt. Ltd. Bangalore, India [18]. Raw reads produced by Illumina technology were filtered by using NGS QC toolkit v2.1 [19]. Filtered sequencing reads was assembled by SOAPdenovo v1.05 [20] (Table S1 and Table S2). Further, all filtered Illumina short reads were used to fill the gaps (Ns, any nucleotide represented by “N”) within the 17 scaffolds of earlier assembled Roche’s 454 data [21] by using Gap Closer v1.10 [22]. Furthermore, all 17 scaffolds (gap filled) were analyzed for the redundancy with the help of BLASTn [23]. Two redundant scaffolds of length 3008 bp and 2543 bp removed from this assembly set. Out of 15 scaffolds left, only one scaffold of 1,404,418 bp length had 811 Ns and these Ns were filled manually by aligning the contigs generated by SOAPdenovo v1.05 assembly (Table 1) with BLASTn. To determine the arrangement of the contigs in the genome of strain SJ98, these 15 contigs were aligned to the genome of *Burkholderia sp.* YI23 by using r2cat software [24]. PCR primers were designed from the ends of the contigs. PCR reactions were carried out to fill the gaps between the scaffolds. The standard PCR reaction mix, 25 µl containing 100 ng genomic DNA, 0.2 mM of each primer, 2.5 µl of 10×PCR buffer, 1 µl of 10 mM dNTPs mix, and 1.25 U of Pfu DNA polymerase (Fermentas, USA). The thermocycler program used for amplification was the following: (i) initial denaturation at 95°C for 5 min; (ii) 10 cycles of denaturation at 95°C for 1 min, primer annealing less than 2–4°C from the mean temperature for each primer sets for 15 sec and fragment amplification at 72°C for 1.5–2.5 min. A final extension was performed for 10 min at 72°C. Only two contigs have been joined by Sanger’s sequencing, finally 7.89-Mb genome draft containing 14 contigs was obtained (Table 1).

**Table 1:** Genome assembly results of *Burkholderia* sp. SJ98.

Genomeassembly	Sequences	Size (bp)	N 50	Ns	GC (%)
**Assembly-1** *	17	7,894,128	1,315,287	58,174	62.23
**Assembly-2** **	17	7,884,563	1,314,594	811	62.68
**Assembly-3** ***	14	7,878,727	1,314,594	0	62.68

* Scaffolds produced by assembly of Roche’s 454 FLX data.

** Sequences (16 contigs and 1 scaffold) produced after gap filling of Assembly-1 by Illumina GA IIX data.

*** Contigs produced after the finishing of Assembly-3 (Sanger’s sequencing and manually by BLAST), final assembly.

Illumina filtered reads were aligned to the 7.89-Mb draft genome by using BWA v 0.6.1 [25] and Samtools v0.1.18 [26] software. All aligned and unaligned reads were exported from alignment files (.bam files) by using bam2fastq [27] software.

### Genome Annotation and Phylogenetic Analysis

We annotate draft genome using NCBI Prokaryotic Genomes Automatic Annotation Pipeline (PGAAP) [18] and RNAmmer 1.2 server [28]. All predicted CDS were again mapped to KEGG [29] pathways using KAAS server [30]. KO (KEGG Orthology) assigned proteins were further analyzed for nitroaromatic compounds degradation pathways.

The complete amino acid sequences of a set of 31 phylogenetic marker genes (primarily genes involved in replication, transcription and translation [31]) were extracted from PGAAP annotation of strain SJ98. BLAST search was performed for (Bacterial RNA polymerase beta subunit) RpoB protein sequence (out of 31 marker genes of strain SJ98) against Non-redundant (NR) database of NCBI and top 33 genome of *Burkholderia* spp. (hits with *rpoB* gene of SJ98) were downloaded from NCBI. All 31 phylogenetic markers genes (amino acid sequences) from all 33 *Burkholderia* strains were extracted. These sequences were aligned to generate a maximum-likelihood tree with 1,000 bootstrap replicates using MEGA5.1 program [32]. Sequences from *Pseudomonas putida* strain ND6 [33] were used as an out-group.

### Genome Comparison

Whole genome comparison of strain SJ98 with strain YI23, CCGE 1001, CCGE 1002 and CCGE 1003 was performed using Jspecies program [34]. This program is commonly used for comparing two gnomes, it uses software BLAST [23] and Mummer v3.0 [35]. We have used OFS v1.2 tool [36] to find out chemotaxis gene cluster in the genome of strain SJ98 and all other compared *Burkholderia* strains. OrthologGroup and paralogGroup have been identified using OrthoMLC tool [37].

### Analysis of Chemotaxis Proteins

All predicted MCPs have been manually extracted from the annotated genome, TMHMM Server v2.0 was used to confirm transmembrane helices (in case of strain SJ98) [38]. Multiple sequence alignment of Chemotaxis proteins (i.e. CheA, CheB, CheR and CheZ) and MCPs of strain SJ98, YI23, CCGE 1001, CCGE 1002, CCGE 1003 and *E. coli* was performed with the help of Clustalx v 2.1 [39].

### Genome Visualization

Annotated genome of *Burkholderia* sp. SJ98 was visualized by JBrowse [40], installed at genome web page [16]. Whole genome comparison of strain SJ98 with strain YI23 has been visualized by Mauve v2.3.1 [41] alignment tool.

## Results

### Whole Genome Assembly

Illumina GAIIX paired end technology produced 41,317,534 paired end reads of 72 nucleotide length, covers ∼371 times of 8-Mb genome of *Burkholderia sp.* SJ98, with an average insert length of 191 nucleotides. We have used NGS QC Toolkit v2.1 [19] to filter the Illumina data for high-quality (HQ) (HQ cut off read length for HQ = 70%, cutoff quality score = 20) (Figure S1-A and S1-B) and Vector and Adaptor contaminations, generates a total of 31,618,692 HQ vector filtered paired-end reads and 3,951,104 single-end reads**.** We have used SOAPdenovo and GapCloser software at different hash lengths (i.e. Kmer) to assemble Illumina data, produced best genome assembly at a hash lengths of 49 (Kmer = 49 for SOAPdenovo) and 17 (Kmer = 17 for GapCloser) respectively (Table S1 and S2). A total of 132 contigs of overall 7.493981-Mb size with N50 contig length of 137,686 bp were obtained as *denovo* genome assembly. *De-novo* genome assembly of Roche’s 454 FLX data produced 17 scaffolds of size 7,894,128 bp containing 58,174 gaps (i.e. Ns) with N50 contig length of 1.3-Mb [42]. Illumina reads (filtered) were used to fill the gaps (i.e. Ns) within 17 scaffolds by using GapCloser v1.0, produces 16 contigs of total size 6,480,677 bp and one scaffold of size 1,403,886 bp (containing 811 gaps i.e. Ns). We have removed two redundant contigs of length 3008 bp and 2543 bp from the 16 contigs obtained following gap closing. All 811 gaps (i.e. Ns) within the mentioned scaffold were filled by a stretch of 387 bp taken from Illumina genome assembly (Table 1). A fragment of 522 bp obtained after Sanger’s sequencing was used to join two contigs of length 738,234 bp and 5128 bp respectively. Finally, we made a draft genome of 14 contigs comprising 7.89-Mb with N50 contig length of 1.3-Mb (Table 1). All Illumina filtered data consists of 31,618,692 paired-end reads and 3,951,104 single-end reads were aligned to 7.89-Mb draft genome. A total of 3,438,348 single-end reads (87%) and 30,960,118 paired-end reads (98%) were aligned to all 14 contigs. Unaligned reads were not used further.

Raw sequencing reads of both technologies (i.e. Roch’s 454 and Illumina GAIIX) have been deposited to SRA under the accession number SRP022216. Whole genome project has been deposited at DDBJ/EMBL/GenBank under the accession number AJHK02000000. This version described in this paper is the second version, AJHK02000000.2.

### General Genome Annotation

Whole genome annotation by PGAAP pipeline of NCBI and RNAmmer v1.2 server produced a total of 7,268 predicted coding regions (CDS), 52 tRNA and 3 rRNAs genes. The genome annotation was visualized by JBrowse tool [39], shows the organization of genes in the genome. The predicted proteins (7,268; minimum length of 18 amino acids, maximum length of 8,741 amino acids) were searched against the Uniprot database (538,585 proteins) and matches were found for 4,801 proteins at an E value cutoff of 10^−6^. Of these, 3,666 proteins could be mapped to the UniProt database. We found the following gene ontology terms after mapping: biological process, 2812; cellular component, 2,067; molecular function, 3,139.

KO assigned proteins obtained from KAAS server mapping, were checked manually. We identified several important genes involved in variety of xenobiotic compounds degradation (table S3).

All predicted 7,268 CDS were submitted to OrthoMLC server, which identify a total of 90 paralog and 6,127 ortholog groups. Paralogous groups includes genes involved in glycosyl transferase family, oxidoreductase domain containing proteins, putative integrases, putative plasmid stable inheritance protein, methane/phenol/toluene hydroxylases, ATPases, transposases, coenzyme PQQ biosynthesis protein PqqD and putative methanol dehydrogenase-like protein/cytochrome cL XoxG. A total of 6,127 orthologous groups have been identified with their scores.

**Figure 1:**
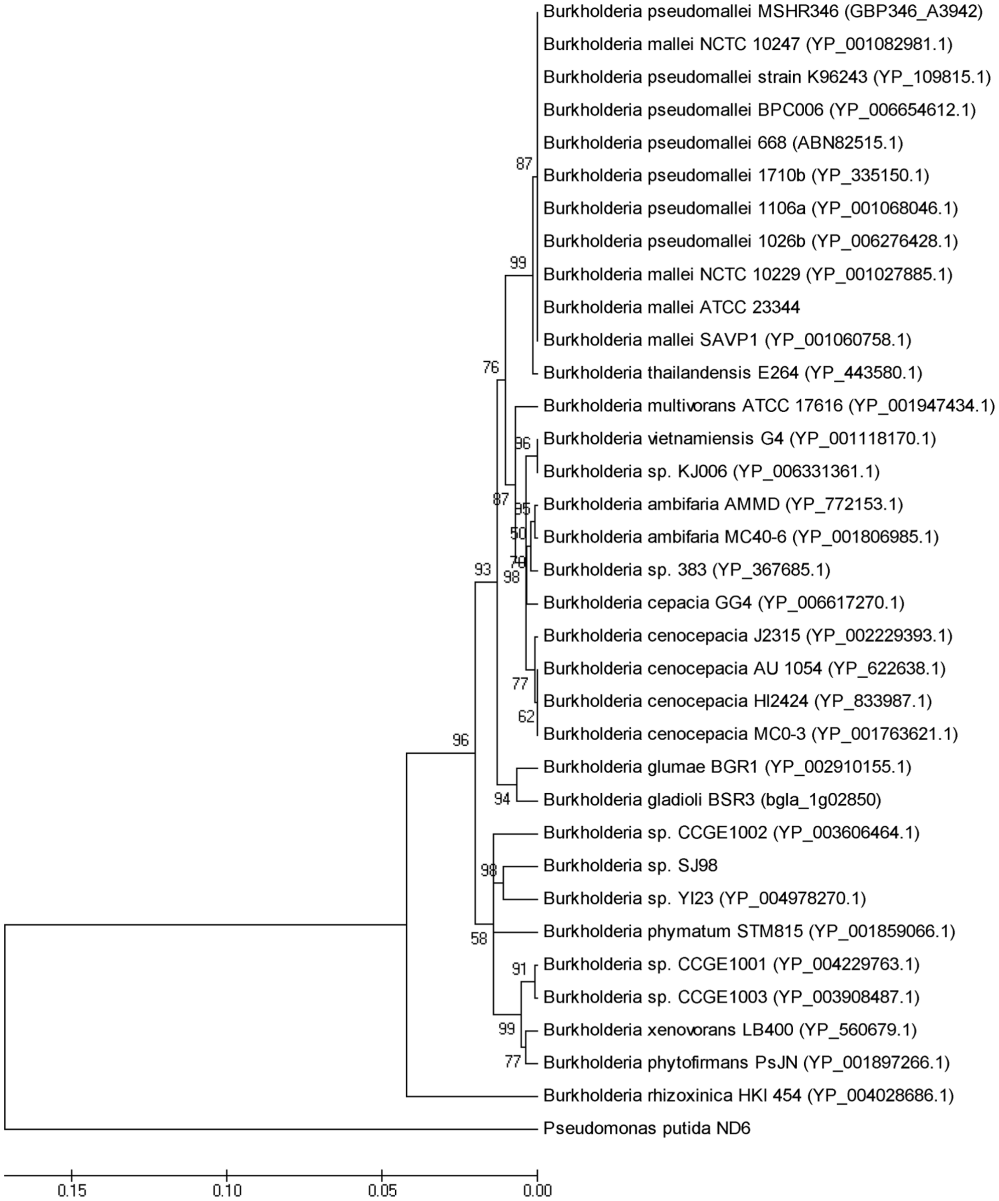
Phylogenomic tree of 33 Burkholderia strains based on amino acid sequences of *rpoB* gene.

**Figure 2:**
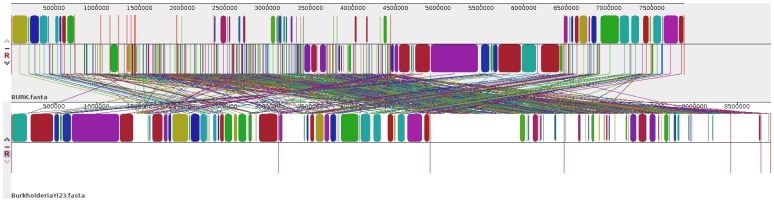
Genome alignment of *Burkholderia* sp. SJ98 and *Burkholderia* sp. YI23.****

Phylogenetic analysis of *rpoB* gene of strain SJ98 with top 33 hits of *Burkholderia* strains reveals its top most similarity with *Burkholderia* sp. YI23. All 31 marker genes of 33 *Burkholderia* strains were aligned to find out the phylogenomic relationship. This phylogenomic interference was drawn on the basis of complete sequences of a set of 31 conserved house-keeping genes that are not considered as horizontal gene transfer [43]. The same sets of genes have been used in past to establish phylogenomic relationships of 106 bacterial and archaeal genomes [44]. As expected, strain SJ98 and strain YI23 are closely related and form their own phylogenetic group (Figure 1). Other closely related strains are CCGE 1001, CCGE 1002 and CCGE 1003, located near the strain SJ98 in phylogenetic tree. Genome characterization these three strains with strain YI23 and SJ98 are given in Table 2. Draft genome of strain SJ98 showed 86.13% BLAST similarity and 88.52% Mummer similarity with genome of strain YI23. Genome comparison between strain SJ98 and YI23 also viewed by Mauve v2.3.1 alignment tool (Figure 2). BLAST similarity values of SJ98 genome with stain CCGE 1001, CCGE 1002 and CCGE 1003 are 76.93%, 77.08% and 76.73% respectively. Further, Mummer similarity values for strains 1001, CCGE 1002 and CCGE 1003 are 84.72%, 84.73% and 84.57% respectively. There is a remarkable difference between compared *Burkholderia* strains, YI23 contains 3 chromosomes and 3 plasmids; CCGE 1001 has 2 chromosomes; CCGE 1002 has 3 chromosomes and 1 plasmid; CCGE 1003 have 2 chromosomes.

**Table 2:** Characterization of *Burkholderia* sp. SJ98, *Burkholderia* sp. YI23, *Burkholderia* sp. CCGE 1001, *Burkholderia* sp. CCGE 1002 and *Burkholderia* sp. CCGE 1003.

Characteristics	*Burkholderia* sp.SJ98	*Burkholderia* sp.YI23	*Burkholderia sp.*CCGE 1001	*Burkholderia sp.*CCGE 1002	*Burkholderia sp.*CCGE 1003
**Length (bp)**	7,878,727	8,896,411	6,833,751	7,884,858	7,043,595
**GC content**	62.68%	63.26%	63.63%	63.27%	63.25%
**No. of protein coding genes**	7,268	7,804	5,965	6,889	5,998
**No. of tRNA genes**	52	64	62	73	63

Whole genome comparison of strain SJ98 with other stains reveals that methane monooxygenase (EC 1.14.13.25), an important enzyme that converts methane to methanol is present in strain SJ98 but absent in YI23, CCGE 1001, CCGE 1002 and CCGE 1003 annotation. ATP dependent Carbamate kinase (EC 2.7.2.2) present in stain SJ98 and CCGE 1003 but absent in stain CCGE 1001, CCGE 1002 and YI23. Catechol 2, 3-dioxygenase, an important enzyme involved in the metabolism of various compounds like Catechol, 4-Chlorocatechol, 3, 4-Dimethylcatechol, 3-Methylcatechol, 4-Methylcatechol, 3-Sulfocatechol and 3-Vinylcatechol is present in stain SJ98 annotation but absent in annotation of YI23, CCGE 1001, CCGE 1002 and CCGE 1003. Creatinase (EC 3.5.3.3) enzyme is also absent in all compared stains but present in strain SJ98.

### Annotation of Chemotaxis Genes

**Figure 3:**
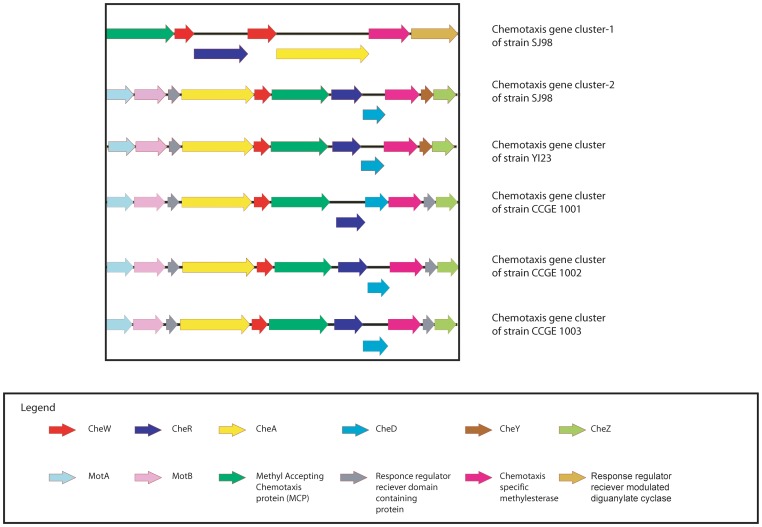
Chemotaxis gene clusters in *Burkholderia* strains SJ98, YI23, CCGE 1001, CCGE 1002 and CCGE 1003.

Strain SJ98 has 19 methyl accepting chemotaxis proteins, all proteins have transmembrane helices with a probability score of >0.8 (computed using TMHMM v2.0), supports the findings (Table 3). In strain SJ98, two chemotaxis gene clusters were found in contig14 and contig13 respectively (Figure 3). First gene cluster have two copies of *CheW* and one copy of *CheR*, *CheA*, *MCP*, *chemotaxis-specific methylesterase* and *response regulator receiver modulated diguanylate cyclase*. Whereas second contains one copy of each gene i.e. *motA*, *motB, response regulator receiver domain-containing protein, CheA, CheW, MCP*, *CheR, CheD, chemotaxis specific protein methylesterase*, *CheY* and *CheZ.* All other MCPs are dispersed in the genome of SJ98. Further, strain YI23 genome was examined for the presence of chemotaxis genes. This analysis revealed the presence of a single chemotaxis gene cluster containing the genes for *motA*, *motB, response regulator receiver domain-containing protein, CheA, CheW, MCP*, *CheR, CheD, chemotaxis specific protein methylesterase*, *CheY* and *CheZ* (Figure 3). Interestingly, chemotaxis gene cluster is also found in other compared strains i.e. CCGE 1001, CCGE 1002 and CCGE 1003 containing same genes as present in YI23 and SJ98 but *CheY* is replaced by a *response regulator receiver domain-containing protein* in these three strains (Figure 3). Complete list of chemotaxis proteins in all compared strains presented in the Table 3. Multiple sequence alignment of the chemotaxis proteins (i.e. CheA, CheB, CheR and CheZ) and MCPs reflects various conserve amino acid regions in all compared *Burkholderia* strains. CheA protein sequences alignment shows conservation of amino acids Ala, Val, Asp, His, Glu, Gly, Glu, Leu, Leu and Leu at the positions 51, 484, 486, 520, 531, 559, 609, 669, 673 and 692 respectively (Figure S2). Various conservation sites were found in the alignment of CheB proteins *i.e*. Leu (197), Arg (282), Ala (293), Asp (311) and Ala (343) (Figure S3). CheR shows two tripeptide (Gly-Glu-Glu 167–169 and Arg-Asn-Val 275–277) and one tetrapeptide (Ile-Tyr-Phe-Asp 279–282) conservation in the alignment (Figure S4). In addition to this, various conserved residues i.e. Ser (165), Asp (195), Ala (204), Tyr (209), Val (251), Gly (306), Glu (309), Arg (90 and 133), Phe (132, 138 and 253) and Leu (201, 258 and 303) are also present in CheR alignment. Protein sequence of CheZ is highly conserved, having one conserved block of 13 residues (Ala-Gln-Asp-Phe-Gln-Asp-Leu-Thr-Gly-Gln-Val-Ile-Lys) in the sequence of all 5 *Burkholderia* strains and *E. coli*. In addition to this, three hexapeptide conservations i.e. Arg-Glu-Leu-Gly-Leu-Asp (44–49), Leu-Leu-Asn-Gly-Pro-Gln (210–215) and Gln-Val-Asp-Asp-Leu-Leu (230–235) also present in the sequences (Figure S5). Multiple sequence alignment of all MCPs have conserved regions from 619- 679 and 692–790 in the alignment (Figure S6).

**Table 3:** Number of *che* gene homologs in *E.coli*, *B*. sp. SJ98, *B*. sp.YI23, *B*. sp. CCGE 1001, *B*. sp. CCGE 1002 and *B*. sp. CCGE 1003.

	Species					
Gene	*E.coli*	*Burkholderia* sp. SJ98	*Burkholderia* sp. YI23	*Burkholderia sp.* CCGE 1001	*Burkholderia sp.* CCGE 1002	*Burkholderia sp.* CCGE 1003
**CheA**	1	2	2	2	2	3
**CheB**	1	4	2	4	3	3
**CheC**	0	1	0	2	1	2
**CheR**	1	3	3	2	2	3
**CheW**	1	5	4	2	2	3
**CheY**	1	2	1	0	0	0
**CheZ**	1	1	1	1	2	1
**MCPs**	4	19	12	22	21	32
**Total**	10	37	25	35	33	47

## Discussion

*Burkholderia* sp. SJ98 is a Gram-negative bacterium responsible for biodegradation of different nitroaromatic compounds. The strain SJ98 is reported to degrade *p*-nitrophenol (PNP), 4-nitrocatechol (4-NC), 3-methyl-4-nitrophenol (3M4NP), *o*-nitrobenzoate (ONB), *p*-nitrobenzoate (PNB), 2-chloro-4-nitrophenol (2C4NP), 4-chloro-2-nitrobenzoate (4C2NB) and 5-chloro-2-nitrobenzoate (5C2NB) as sole source of carbon and energy [7], [10], [11] and transforms *o*-dinitrobenzene, m-dinitrobenzene m-nitrophenol, 2,4-dinitrophenol, 2,5-dinitrophenol 2,6-dinitrophenol, 3,5-dinitrobenzoate 2-chloro-3-nitrophenol (2C3NP) and 2-chloro-4-nitrobenzoate (2C4NB) [8], [9]. This strain is chemotactic towards all above compounds and having bioremediation potential. Thus, we sequenced, assembled and annotate whole genome of strain SJ98 to explore its full potential. Recently, first genome draft of strain SJ98 was published by Kumar *et. al*. [21]. Here, we have again sequenced whole genome of strain SJ98 by Illumina technology and filled the gaps remained in the first genome draft by both Illumina and Sanger’s technique. Assembly of this genome performed in several steps that includes; i) assembly of genome by SOAPdenovo and GapCloser v1.0, ii) number of gaps (i.e. Ns) have been removed using GapCloser and iii) contigs generated by different techniques were joined. After removing redundancy in the assembled contigs, we obtained a draft genome of size 7.87-Mb.

These genome finishing approaches resulted in a robust assembled genome of *Burkholderia* sp. SJ98, which was annotated with PGAAP pipeline. This pipeline combines HMM-based gene prediction methods with a sequence similarity-based approach that combines the comparison of the predicted gene products to the non-redundant protein database, Entrez Protein Clusters, the Conserved Domain Database, and COGs (Clusters of Orthologous Groups) and best choice for annotation in this study [45]. PGAAP have been used for in RefSeq project to improve the annotation of complete microbial genomes. The complete genome annotation of strain SJ98 is available at the NCBI with the accession number AJHK02000000.2 [46].

To establish the phylogenetic relationship of strain SJ98 with existing *Burkholderia* spp., comparative genomics approach was adopted includes the analysis of amino acid sequence of *rpoB* gene and all other housekeeping genes. Gene *rpoB* is highly conserved within the species, so amino acid sequence was taken for phylogenetic comparison of strain SJ98 with closely related 33 *Burkholderia* spp. The analysis (by comparing 31 housekeeping genes) of all 34 *Burkholderia* spp. reveals that strain SJ98 with YI23 together making their own phylogenetic group. Strain YI23 was isolated as fenitrothion (O, O-dimethyl-O-[p-nitro-m-tolyl] phosphorothioate) degrading bacterium from a golf course soil and is able to quickly degrade diverse organophosphorus pesticides [42].

BLAST and Mummer similarity results also showing that strain *Burkholderia* sp. YI23 is closely related to strain SJ98 as compare to CCGE 1001, CCGE 1002 and CCGE 1003. As housekeeping genes remains conserved in the genus, so, location of all 31 housekeeping genes have been identified (Sheet S1) in stains SJ98 and YI23 to locate the contigs of stain SJ98 with respect to stain YI23. This analysis gives the idea that contigs 4, contigs 13, contigs 14 and contigs 1 may represent chromosome 1 in strain SJ98.

Whole genome analysis indicates that all the compared strains SJ98, YI23, CCGE 1001, CCGE 1002 and CCGE 1003 have genes of Glycolysis and Gluconeogenesis, TCA cycle, Chitin and N-acetylglucosamine utilization, Calvin-Benson cycle, Photorespiration (oxidative C2 cycle), Entner-Doudoroff Pathway and Pentose phosphate pathway. Genes for the utilization of several carbohydrates like Lactate, D-ribose, L-arabinose, Maltose and Maltodextrin, Chitin and N-acetylglucosamine, Maltose and Maltodextrin, 2-Ketogluconate, L-fructose, Inositol and Xylose. Comparative studies of central metabolic pathway genes indicate that these organisms having the similar type of physiology under the normal environment. Presence of genes like methane monooxygenase, catechol 2,3 dioxygenase and creatinase, that are absent in other strains (e.g., YI23, CCGE 1001, CCGE 1002, CCGE 1003) indicates that strain SJ98 have a higher catabolic potential than other compared strains.

Chemotaxis proteins (CheA, CheB, CheC, CheR, CheW, CheY and CheZ) and MCPs are necessary proteins for the bacterial chemotaxis. Chemotaxis protein CheA is involved in the transmission of sensory signals from the chemoreceptors to the flagellar motors. CheA is autophosphorylated; it can transfer its phosphate group to either CheB or CheY. CheA have three functional domains: one for interaction with CheB and CheY, a second for regulating phosphorylation and controlling the stability of the protein, and a third for receiving input signals regulating CheA activity [47]. CheB is phosphorylated by CheA [48]. CheC involved in restoring normal CheY-P levels by dephosphorylating CheY-P. CheR has S-adenosylmethionine-dependent methyltransferase activity. CheW Involved in the transmission of sensory signals from the chemoreceptors to the flagellar motors. It physically bridges CheA to the MCPs (methyl-accepting chemotaxis proteins) to allow regulated phosphotransfer to CheY and CheB [49].

CheY is phosphorylated by CheA or acetylated by acetyl-CoA synthetase, depending on which acetate metabolism pathway is available. The major acetylation site seems to be Lys-92. CheY is dephosphorylated (inactivated) by CheZ [2], [50], [51], [52], [53]. MCPs (methyl accepting chemotaxis proteins) are a family of bacterial receptors that mediate chemotaxis to several signals, responding to changes in the concentration of attractants and repellents in the environment by altering swimming behavior [54]. Environmental diversity gives rise to diversity in bacterial signaling receptors, and consequently there are many genes encoding MCPs [55].

Pandey *et. al*., (2011) has reported that strain SJ98 is chemotactic towards the NACs [8]. They have observed that strain SJ98 does not shows chemotaxis towards the 4C2NP (not degraded or co-metabolized by *Burkholderia* sp. SJ98). All 19 MCPs of strain SJ98 were ascertained for having transmembrane helices and confirmed that they might be involved in the process of chemotaxis. During our analysis, chemotaxis gene cluster have been found1 in the genome of this important bacterium. Gene’s *motA* and *motB* are required for the generation of torque during the flagellar movement of a bacterium [56]. Presence of *motA* and *motB* genes within the chemotaxis gene cluster in SJ98 reflects that they might be actively participating in the chemotaxis by forming a functional flagellar motor as in case of *E. coli* [57]. In the strain YI23, presence of chemotaxis genes along with *motA* and *motB* in a single gene cluster indicates that this bacterium might have the chemotaxis property which has not been studied so far. Interestingly, arrangement of chemotaxis genes in the cluster of strain SJ98 (i.e. cluster-2) and other compared strains, YI23, CCGE 1001, CCGE 1002 and CCGE 1003 is same (Figure 3). This indicates the conservation of chemotaxis gene cluster between the strain SJ98 and other compared strains. This gene cluster is present at complimentary stand in case of all compared *Burkholderia* spp. but present at forward strand in case of SJ98 only. Chemotaxis gene *cheY* is not present in strain CCGE 1001, CCGE 1002 and CCGE 1003 but a response regulator receiver domain containing protein is instead present at that location in the cluster. This response regulator may function like *cheY*. In past, genome sequence and annotation of genus Geobactor spp. reveals the chemotaxis genes, further the chemotaxis activity was ascertained by experimental approaches [58]. So, such type of study can be done in case of strain YI23 in near future. Strain CCGE 1002 also have 190 CDS related to aromatic compound metabolism but the chemotaxis property in this strain is not yet reported [59]. Presence of genes catechol 1,2 dioxygenase, hydroxyquinol 1,2-dioxygenase, aromatic-ring-hydroxylating dioxygenase, 4-hydroxybenzoate 3-monooxygenase and 2-nitropropane dioxgenase in stain CCGE 1001 and CCGE 1003 indicates that these strains may involve in the degradation of xenobiotics. Although, chemotaxis property of strain CCGE 1001, CCGE 1002 and CCGE 1003 is not yet reported but presence of *che* genes in the genome of these microbes provides the way to discover this phenomenon in these microbes.

Multiple sequence alignment of the amino acid sequence of the *che* genes (i.e. CheA, CheB, CheR and CheZ) reveals that there is high sequence homology between the strain SJ98 other compared strains specially in case of CheR and CheZ. It was also observed that *E. coli* K12 *che* genes were showing homology with the mentioned *che* genes of both the strains SJ98 and strain YI23. Multiple sequence alignment of all MCPs of *E.* coli K12, *Burkholderia* strains i.e. YI23, CCGE 1001, CCGE 1002, CCGE 1003 and SJ98 shows the conserved regions, that indicates the evolution of these genes from *E. coli* to genus *Burkholderia*.

### Conclusions

In this study complete genome of nitroaromatic and chloronitroaromatic compounds degrading bacterium *Burkholderia* sp. SJ98 has been explored for the identification of various genes involved in the chemotaxis and NACs and NACs degrading pathways. Conserved regions identified in the multiple sequencing alignments of all MCPs of *E.* coli K12 and five compared *Burkholderia* strains reflects the evolutionary relationships between *E. coli* and *Burkholderia* spp. The comparative genomics study provides the insight that strain SJ98 is very close to a newly characterized *Burkholderia* sp. strain YI23. Current study indicates that *Burkholderia* sp. SJ98 could be used as a model system to further analyze the molecular mechanisms of chemotaxis towards nitroaromatic compounds, which is still not very well studied.

## Supporting Information

Figure S1 **(A):** Average quality score of Illumina forward reads. **(B):** Average quality score of Illumina reversed reads.
(TIF)

Figure S2 **Multiple sequence alignment of CheA proteins.**
(TIF)

Figure S3 **Multiple sequence alignment of CheB proteins.**
(TIF)

Figure S4 **Multiple sequence alignment of CheR proteins.**
(TIF)

Figure S5 **Multiple sequence alignment of CheZ proteins.**
(TIF)

Figure S6 **Multiple sequence alignment of MCPs.**
(TIF)

Table S1 ***denovo*** **genome assembly of Illumina data with SOAPdenovo v1.05 at different hash length (K).**
(DOC)

Table S2 **Closing of gaps for best scaffold set (i.e. K = 49) at different hash length (K) by GapCloser v1.0 software.**
(DOC)

Table S3 **Genes identified in the genome of** ***Burkholderia*** **sp. SJ98, involved in the degradation of different xenobiotic compounds.**
(DOC)

Sheet S1 **Location of 31 housekeeping genes in the genome of** ***Burkholderia*** **sp. SJ98 and** ***Burkholderia*** **sp. YI23.**
(XLS)
