# Supplementary figures and images for: Genome Annotation of Burkholderia sp. SJ98 with Special Focus on Chemotaxis Genes
Source: PLoS One. 2013 Aug 5;8(8):e70624. doi: 10.1371/journal.pone.0070624 (PMC3734258; doi:10.1371/journal.pone.0070624)

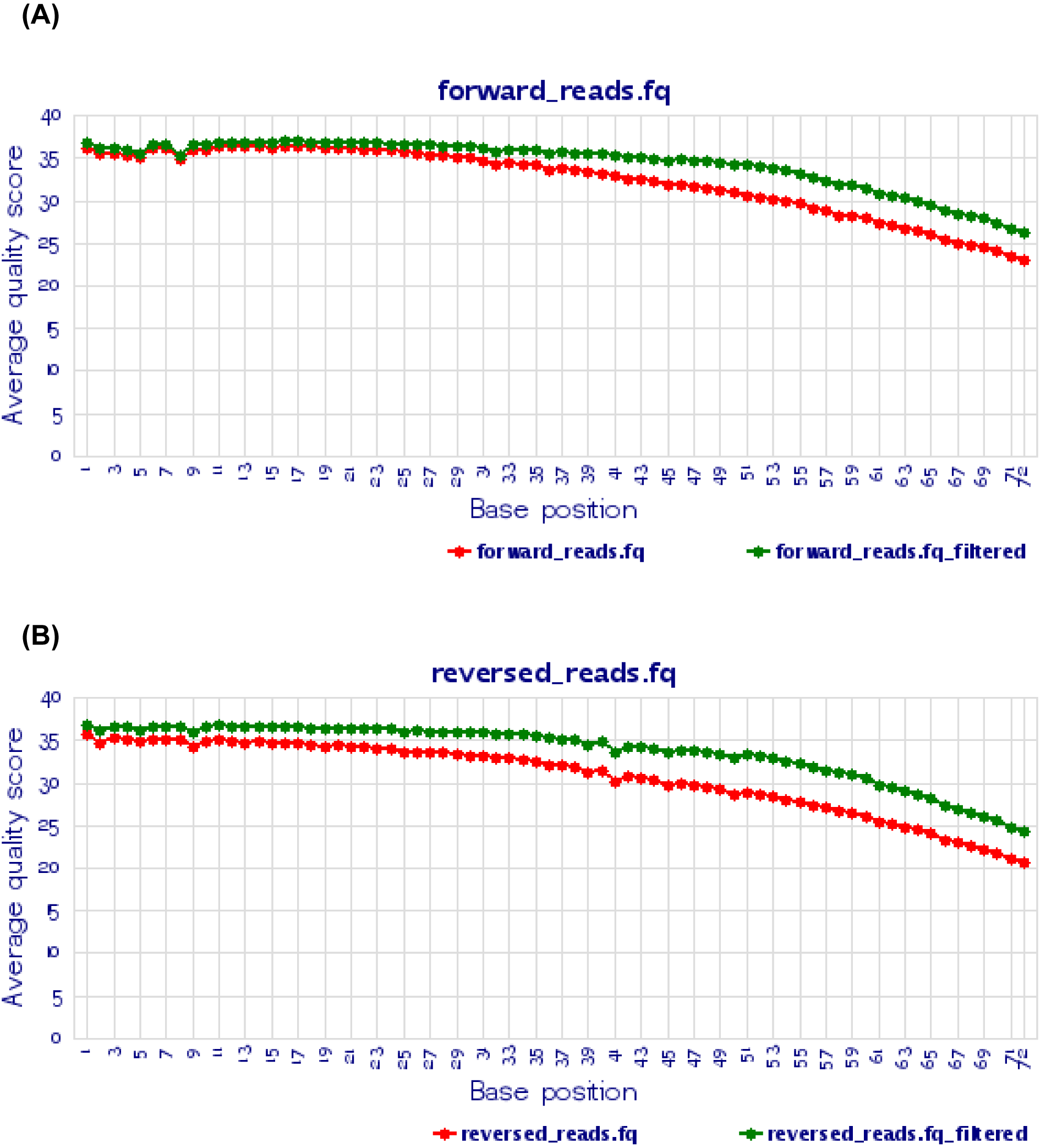

Supplement: Figure S1 — (A): Average quality score of Illumina forward reads. (B): Average quality score of Illumina reversed reads. (TIF) [file pone.0070624.s001.tif]

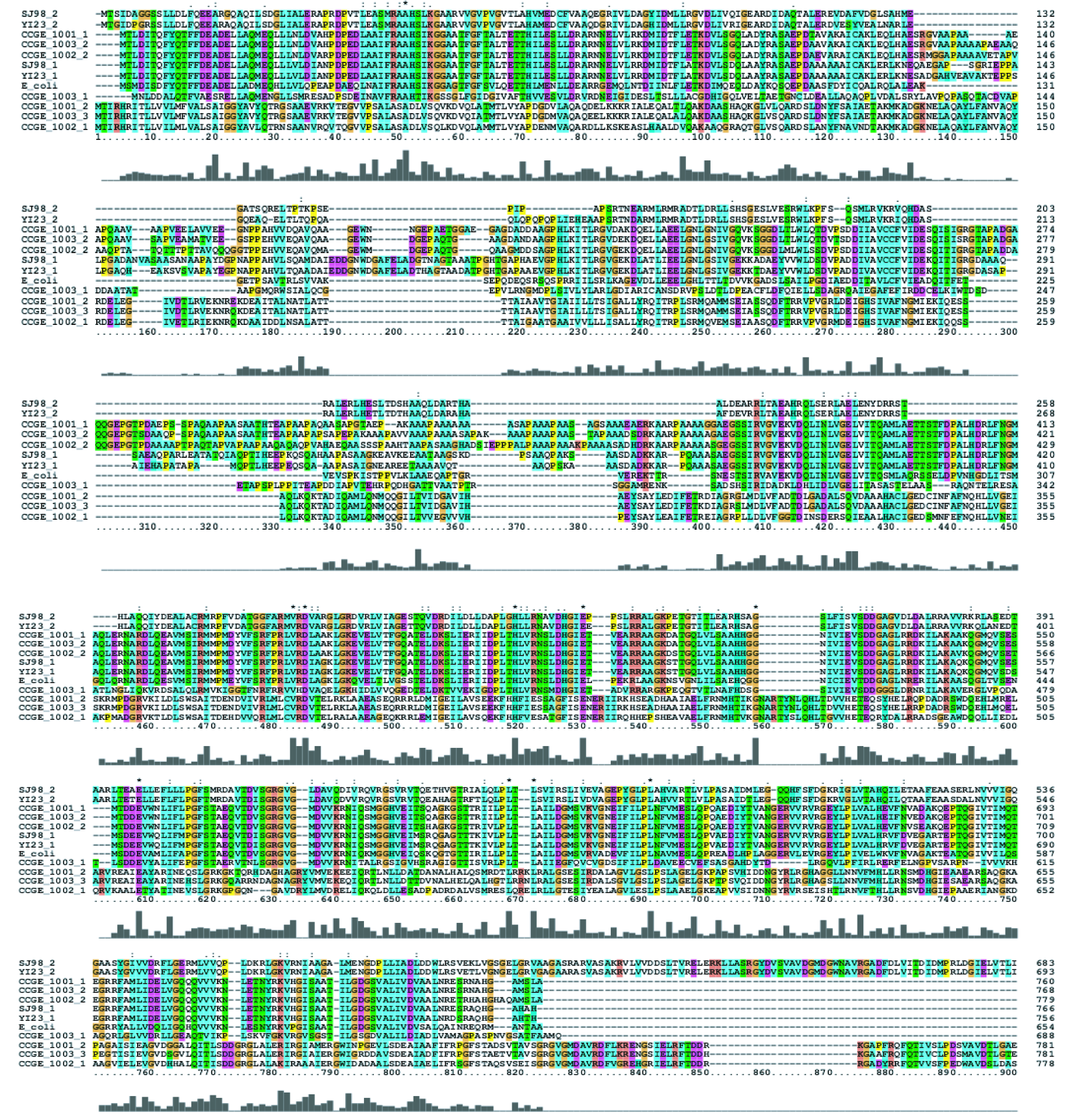

Supplement: Figure S2 — Multiple sequence alignment of CheA proteins. (TIF) [file pone.0070624.s002.tif]

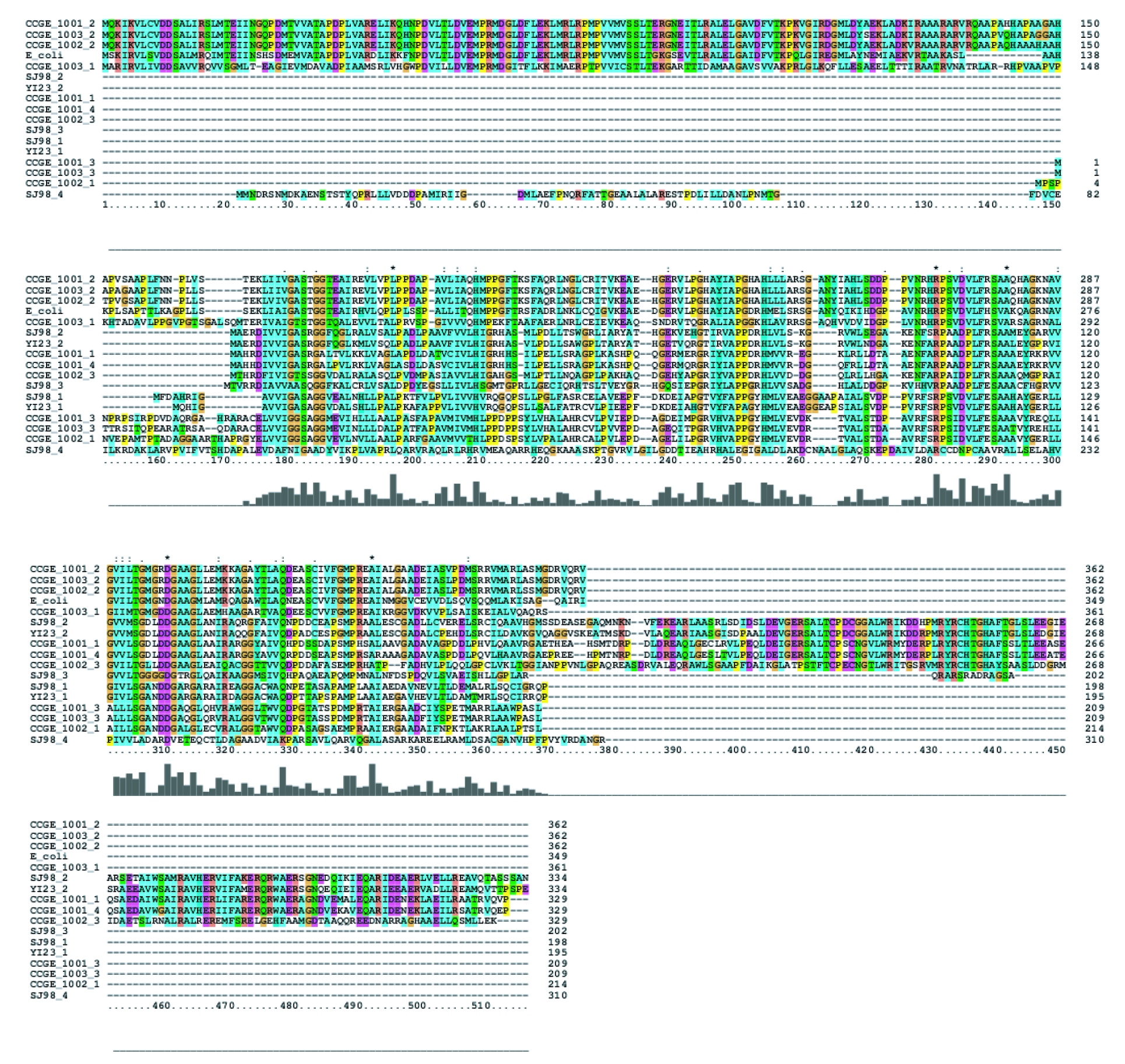

Supplement: Figure S3 — Multiple sequence alignment of CheB proteins. (TIF) [file pone.0070624.s003.tif]

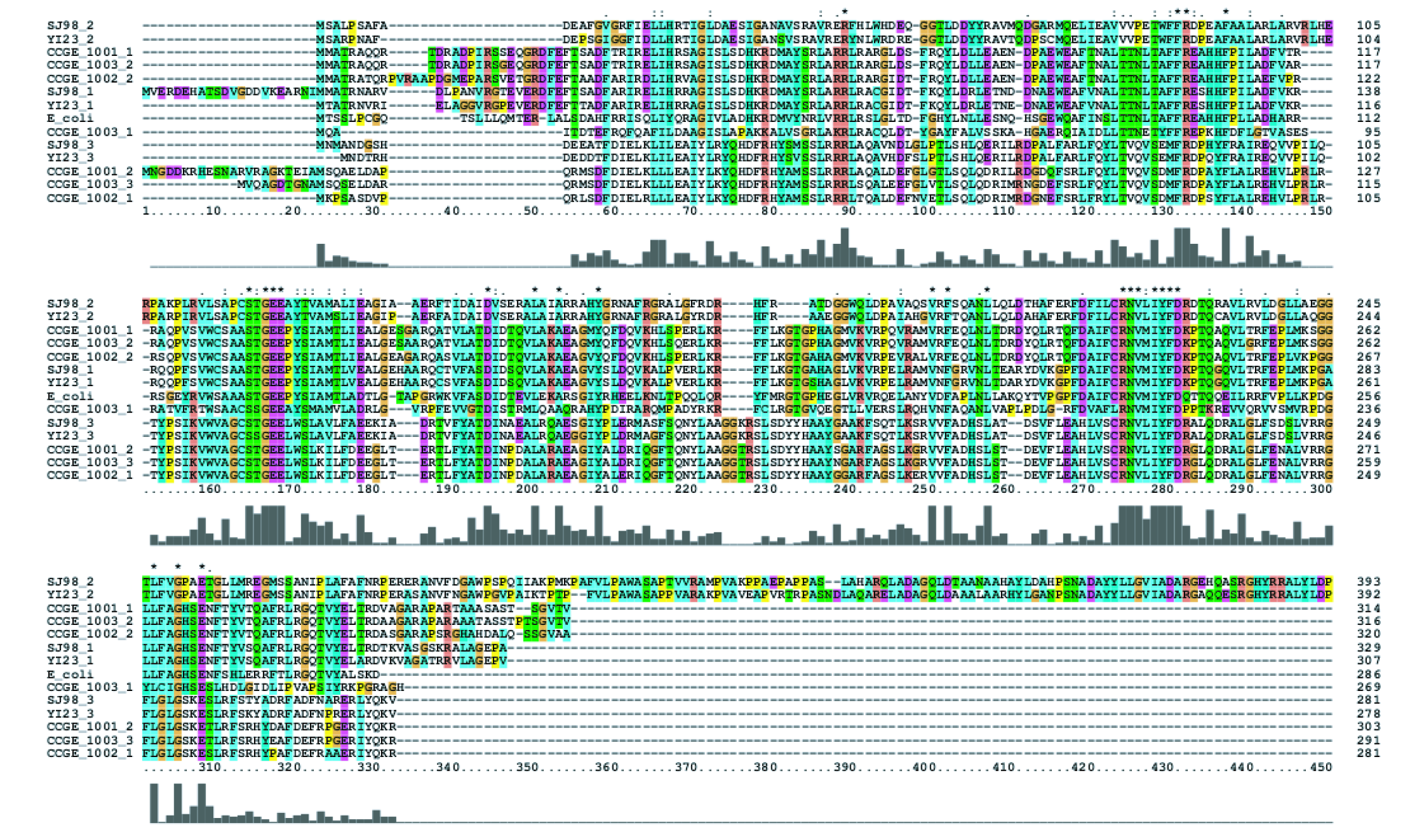

Supplement: Figure S4 — Multiple sequence alignment of CheR proteins. (TIF) [file pone.0070624.s004.tif]

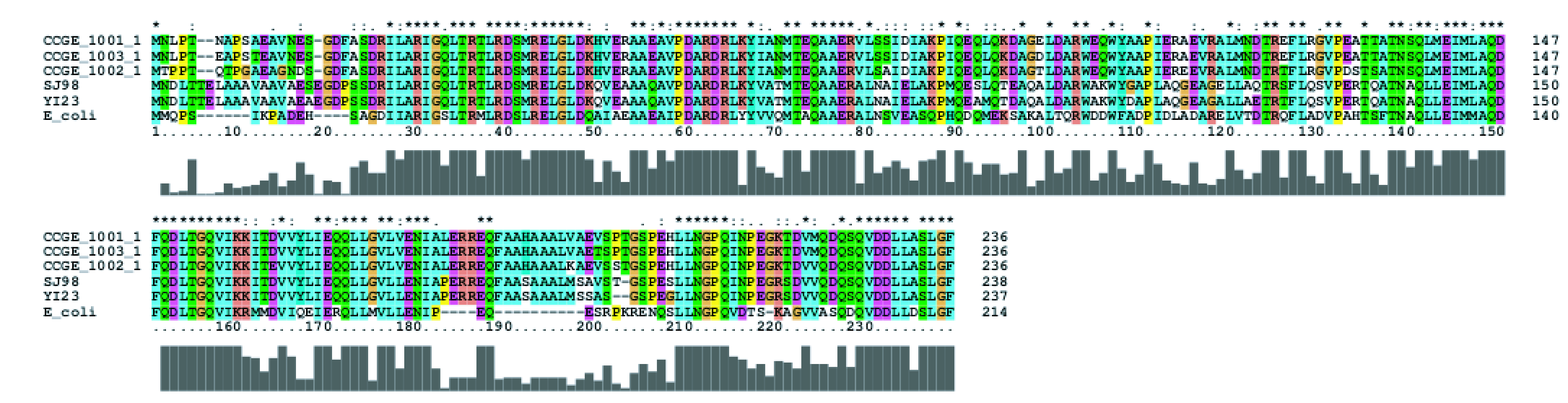

Supplement: Figure S5 — Multiple sequence alignment of CheZ proteins. (TIF) [file pone.0070624.s005.tif]

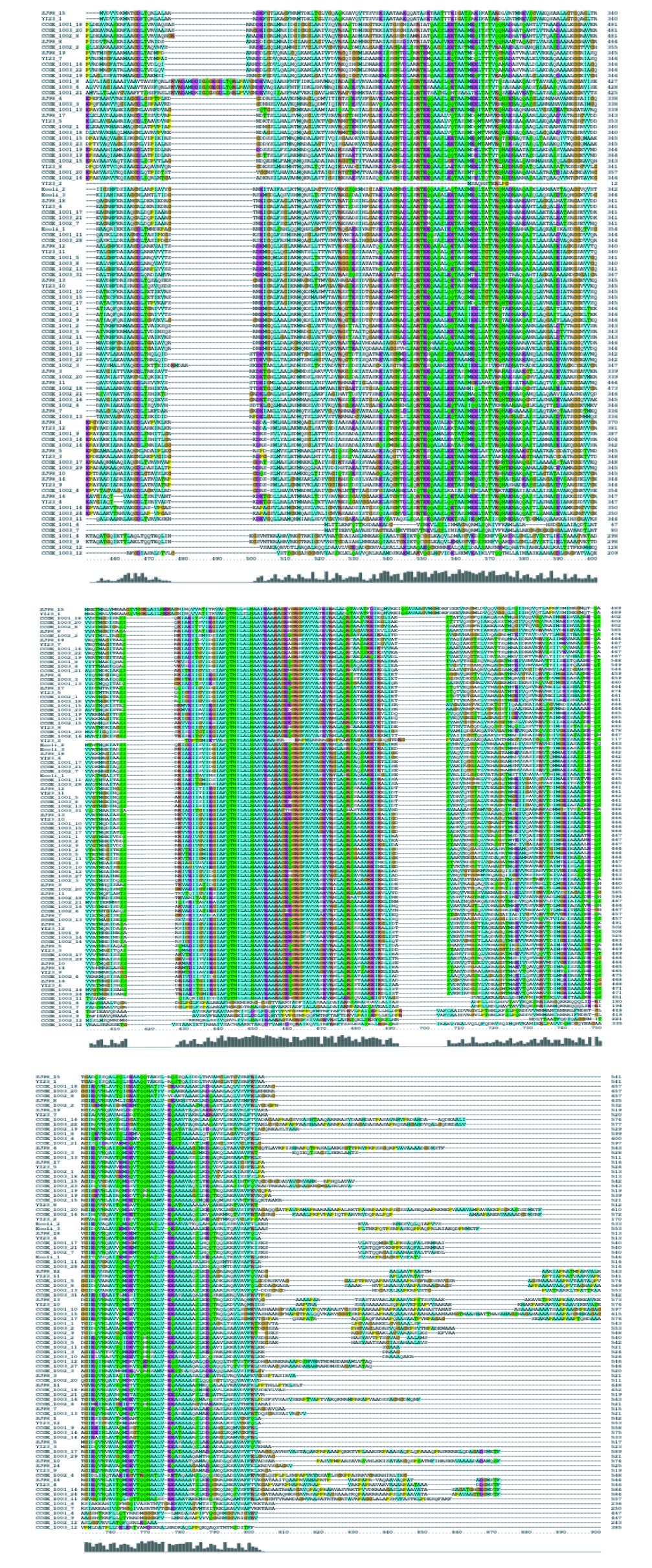

Supplement: Figure S6 — Multiple sequence alignment of MCPs. (TIF) [file pone.0070624.s006.tif]
