# Supplementary material for: Genome Annotation of Burkholderia sp. SJ98 with Special Focus on Chemotaxis Genes
Source: PLoS One. 2013 Aug 5;8(8):e70624. doi: 10.1371/journal.pone.0070624 (PMC3734258; doi:10.1371/journal.pone.0070624)
Supplement: Table S1 — denovo genome assembly of Illumina data with SOAPdenovo v1.05 at different hash length (K). (DOC) [file pone.0070624.s007.doc]

**Table S1:** *denovo* genome assembly of Illumina data with SOAPdenovo v1.05 at different hash lengths (K).

| **SOAPdenovo1.05** | | | | | | | | | | |
| --- | --- | --- | --- | --- | --- | --- | --- | --- | --- | --- |
| K | Contigs | N 50 | Genome size(Mb) | GC (%) | Scaffolds | N 50 | Genome size(Mb) | Ns | Ns (%) | GC (%) |
| 45 | 3716 | 21361 | 8.00 | 62.66 | 146 | 160044 | 7.63 | 14389 | 0.19 | 62.62 |
| 47 | 2866 | 19302 | 7.96 | 62.67 | 142 | 137572 | 7.48 | 12648 | 0.17 | 62.64 |
| **49** | **2299** | **18852** | **7.93** | **62.68** | **132** | **137659** | **7.48** | **12527** | **0.17** | **62.65** |
| 51 | 2030 | 18122 | 7.91 | 62.68 | 134 | 137629 | 7.45 | 13950 | 0.19 | 62.64 |
| 53 | 1967 | 16579 | 7.91 | 62.69 | 138 | 109554 | 7.53 | 15099 | 0.20 | 62.59 |
