# Supplementary material for: Genome Annotation of Burkholderia sp. SJ98 with Special Focus on Chemotaxis Genes
Source: PLoS One. 2013 Aug 5;8(8):e70624. doi: 10.1371/journal.pone.0070624 (PMC3734258; doi:10.1371/journal.pone.0070624)
Supplement: Table S2 — Closing of gaps for best scaffold set (i.e. K = 49) at different hash length (K) by GapCloser v1.0 software. (DOC) [file pone.0070624.s008.doc]

**Table S2:** Closing of gaps for best scaffold set (i.e. K=49) at different hash length (K) by GapCloser v1.0 software.

| **K** | **N 50** | **Genome size(Mb)** | **Ns** | **GC (%)** |
| --- | --- | --- | --- | --- |
|  | 137,659 | 7.48 | 12527 | 62.65 |
| **17** | **137,686** | **7.494** | **0** | **62.75** |
| 19 | 137,707 | 7.493 | 65 | 62.75 |
| 21 | 137,704 | 7.493 | 56 | 62.75 |
| 23 | 137,704 | 7.493 | 142 | 62.75 |
