# Supplementary material for: Genome Annotation of Burkholderia sp. SJ98 with Special Focus on Chemotaxis Genes
Source: PLoS One. 2013 Aug 5;8(8):e70624. doi: 10.1371/journal.pone.0070624 (PMC3734258; doi:10.1371/journal.pone.0070624)
Supplement: Table S3 — Genes identified in the genome of Burkholderia sp. SJ98, involved in the degradation of different xenobiotic compounds. (DOC) [file pone.0070624.s009.doc]

**Table S3:** Genes identified in the genome of *Burkholderia* sp. SJ98, involved in the degradation of different xenobiotic compounds.

| **S.No.** | **Xenobiotic compound** | **Genes involved in degradation pathway** |
| --- | --- | --- |
| 1. | Fluorobenzoate | Benzoate/toluate 1,2-dioxygenase subunit alpha, Dihydroxycyclohexadiene carboxylate dehydrogenase, Catechol 1,2-dioxygenase, Cycloisomerase, Nitrile hydratase, Carboxymethylenebutenolidase, Muconate cycloisomerase |
| 2. | Chlorocyclohexane and chlorobenzene | Muconate cycloisomerase, Haloalkane dehalogenase, Haloacetate dehalogenase, 2-haloacid dehalogenase, Carboxymethylenebutenolidase, Benzoate/toluate 1,2-dioxygenase subunit alpha, Catechol 1,2-dioxygenase, Muconate cycloisomerase |
| 3. | Xylene | 2-keto-4-pentenoate hydratase, 2-keto-4-pentenoate hydratase, 4-hydroxy 2-oxovalerate aldolase, Acetaldehyde dehydrogenase (acetylating), Benzoate/toluate 1,2-dioxygenase subunit alpha, Dihydroxycyclohexadiene carboxylate dehydrogenase |
| 4. | Dioxan | 2-keto-4-pentenoate hydratase, 4-hydroxy 2-oxovalerate aldolase, Acetaldehyde dehydrogenase (acetylating), Biphenyl 2,3-dioxygenase subunit alpha, Salicylate hydroxylase. |
| 5. | Styrene | Phenylacetaldehyde dehydrogenase, Amidase,Homogentisate 1,2-dioxygenase, Maleylacetoacetate isomerase, Fumarylacetoacetase, Aliphatic nitrilase, Nitrile hydratase |
| 6. | Limonene and pinene | Aldehyde dehydrogenase (NAD+), Enoyl-CoA hydratase |
| 7. | Chloroalkane and Chloroalkene | Glutathione-independent formaldehyde dehydrogenase, Haloalkane dehalogenase, Haloacetate dehalogenase, 2-haloacid dehalogenase, Carboxymethylenebutenolidase, Aldehyde dehydrogenase (NAD+), Benzoate/toluate 1,2-dioxygenase subunit alpha |
| 8. | Toluene | Benzoate/toluate 1,2-dioxygenase subunit alpha, Catechol 1,2 dioxygenase, Muconate cycloisomerase, Carboxymethylenebutenolidase |
| 9. | Benzoate | Benzoate/toluate 1,2-dioxygenase subunit alpha, Dihydroxycyclohexadiene carboxylate dehydrogenase, Catechol 1,2-dioxygenase, Muconate cycloisomerase, Haloalkane dehalogenase, Haloacetate dehalogenase, 2-haloacid dehalogenase, Carboxymethylenebutenolidase, Protocatechuate 3,4-dioxygenase, alpha subunit, 2-keto-4-pentenoate hydratase, 4-hydroxy 2-oxovalerate aldolase, Acetaldehyde dehydrogenase (acetylating), 3-carboxy-cis,cis-muconate cycloisomerase, 4-carboxymuconolactone decarboxylase |
| 10. | Aminobenzoate | Nitrile hydratase, Amidase, Enoyl-CoA hydratase |
| 11. | Ethylbenzene | Ethylbenzene dioxygenase alpha and beta subunit |
